# The Action of Phosphorus as a Stimulant

**Published:** 1875-04

**Authors:** John Brunton

**Affiliations:** Fellow and Counselor of the Medical Society of London, etc.


### THE ACTION OF PHOSPHORUS AS A STIMULANT.

BY JOHN BRUNTON, M. A., M. D.,

*Fellow and Counselor of the Medical Society of London, etc.*

Nearly if not all the writers upon therapeutic action of phosphorus are agreed in this: that in certain conditions of extreme physical depression or exhaustion, the action of this drug, in full doses, is of exceeding value. Patients who, to all appearance, are sinking rapidly, are stimulated and tided over their difficulty. In those eruptive fevers which have a definite term, and towards the close of whose course, just before the turn, the exhaustive depression is very great, phosphorus in stimulating doses acts like a charm.

The difficulties that heretofore have been met with in the administration of this drug, as regards stability, solution, absorption and taste, and the consequent uncertainty accruing thereto, have thrown it into desuetude; but now that convenient and certain formulæ exist, a fair opportunity has arisen for testing the marvelous powers of this agent. On this account, I take an early opportunity of testifying to its powers.*

* For full information on the subject the reader will do well to refer to the recently published work, "Free Phosphorus in Medicine," by J. Ashburton Thompson, pp. 32, 33, and 165.

Five weeks ago, Mrs. M—, aged forty-seven, was in the twelfth day of typhus fever. Her depression was very great. She lay in bed cold, nearly pulseless, semi-unconscious and with difficulty aroused, quite unable to move in bed; in short, she appeared moribund. One-twelfth of a grain of phosphorus was administered every two hours, with marvelous effect. The revivifying power was very marked, by return of heat, pulse, consciousness, and general power. Next day she had the turn, and has recovered admirably.

J. C—, aged forty, a railway goods guard, from Peterborough, came to me on the 12th August, suffering from diarrhœa, which at that time and season was very prevalent. In a day or two he had the appearance of having something more than mere diarrhœa.—He became too ill to get out, and took to his bed. On examination, I found the characteristic rose-colored spots of typhoid fever, and he had all the other symptoms present. His fever continued gradually to increase till the 29th August, when he said to me, "I am going to die"—and he looked like it. His conjunctivæ were injected, his breath cold, his skin cold and clammy; pulse forty-eight, very weak and compressible; voice whispering; temperature 96.4°. He was in a condition of extreme depression. I at once administered phosphorus, in doses of one-twelfth of a grain, every two hours, and I was surprised to find on my next visit, eighteen hours after, when he had taken three-quarters of a grain of the drug, that he had quite revived. His skin was comfortably warm, eyes not so suffused, voice more natural; pulse 72; temperature 99°. I immediately stopped the phosphorus and gave nitric acid. Since that he has gone on prosperously, and is now convalescent.

Of course, many more cases are needed to form a good induction, but I record these two for the purpose of inducing others to try the remedy. Heroic doses need careful watching, and I am sure the formula appended is most stable and active, and it is not unpalatable. I do not think I should care to go beyond one, or at most two grains of the drug, divided over two days. The second case is a good illustration that the drug is to be suspended when the desired effect is produced.

*Formula.*—Etherial tinct. phosp. (gr. $\frac{1}{3}$ to ʒ i.), ʒ iii.; spt. vin. rect., ℥ ss.; glycerin. anhydr, ad ℥ iss. One teaspoonful as a dose. —*London Lancet.*

